# Transcatheter mitral valve replacement versus redo surgery for mitral prosthesis failure: A systematic review and meta-analysis

**DOI:** 10.3389/fcvm.2022.1058576

**Published:** 2023-01-18

**Authors:** Jiawei Zhou, Yuehuan Li, Zhang Chen, Haibo Zhang

**Affiliations:** Department of Cardiac Surgery, Beijing Anzhen Hospital, Capital Medical University, Beijing, China

**Keywords:** redo, surgical mitral valve replacement, mitral prosthesis failure, transcatheter mitral valve replacement (TMVR), meta-analysis

## Abstract

**Background:**

Transcatheter mitral valve replacement (TMVR) has emerged as an alternative to redo surgery. TMVR compared with redo surgical mitral valve replacement (SMVR) in patients with mitral prosthesis failure remains limited. In this study, we performed a meta-analysis to assess the outcomes of TMVR (including valve-in-valve and valve-in-ring) versus redo surgery for mitral prosthesis failure.

**Methods:**

We comprehensively searched the PubMed, Embase, and Cochrane library databases according to predetermined inclusion and exclusion criteria, and then we extracted data. We compared the outcomes of TMVR and redo SMVR for mitral prosthesis failure in terms of the in-hospital mortality, stroke, renal dysfunction, vascular complication, pacemaker implantation, exploration for bleeding, paravalvular leak, mean mitral valve gradient, 30-day mortality, and 1-year mortality.

**Results:**

Nine retrospective cohort studies and a total of 3,038 patients were included in this analysis. Compared with redo SMVR for mitral prosthesis failure, TMVR was associated with lower in-hospital mortality [odds ratios (OR): 0.44; 95% confidence interval (CI): 0.30–0.64; *P* < 0.001], stroke (OR: 0.44; 95% CI: 0.29–0.67; *P* = 0.0001), renal dysfunction (OR: 0.52; 95% CI: 0.37–0.75; *P* = 0.0003), vascular complication (OR: 0.58; 95% CI: 0.43–0.78; *P* = 0.004), pacemaker implantation (OR: 0.23; 95% CI: 0.15–0.36; *P* < 0.00001), and exploration for bleeding (OR: 0.24; 95% CI: 0.06–0.96; *P* = 0.04). Conversely, redo SMVR had lower paravalvular leak (OR: 22.12; 95% CI: 2.81–174.16; *P* = 0.003). There was no difference in mean mitral valve gradient (MD: 0.04; 95% CI: −0.47 to 0.55; *P* = 0.87), 30-day mortality (OR: 0.65; 95% CI: 0.36–1.17; *P* = 0.15), and 1-year mortality (OR: 0.96; 95% CI: 0.63–1.45; *P* = 0.84).

**Conclusion:**

In patients with mitral prosthesis failure, TMVR is associated with lower in-hospital mortality and lower occurrence of postoperative complications, except for paravalvular leak. TMVR offers a viable alternative to the conventional redo surgery in selected patients.

## 1. Introduction

Mitral bioprostheses replacement or implantations of valve reconstructive rings provide benefit to patients due to better hemodynamics and shorter anticoagulation time. However, mitral bioprostheses and reconstructive rings might fail within a few years since surgery (1, 2). Up to 35% of patients who have had mitral valve surgery may need to undergo redo surgery (3). Redo surgical mitral valve replacement (SMVR) is associated with a greater operative risk and high mortality (4–6). Recently, transcatheter mitral valve-in-valve or valve-in-ring replacement has emerged as a minimally invasive option (7–9). Data comparing the outcomes of this approach with those of open redo surgery are limited (10). Herein, we performed a systematic review and meta-analysis to provide a more comprehensive review of the clinical and echocardiographic outcomes of transcatheter mitral valve replacement (TMVR) (including valve-in-valve and valve-in-ring) compared with redo SMVR for the treatment of degenerated mitral prosthesis. The aim of the present study was therefore to evaluate the safety and efficacy of TMVR compared with redo SMVR for mitral prosthesis failure.

## 2. Methods

### 2.1. Literature search

The systematic review and meta-analysis were performed in accordance with the Preferred Reporting Items for Systematic Reviews and Meta-Analyses (PRISMA) guidelines. Systematic search using PubMed, Embase, and Cochrane Library databases was independently carried out by two authors to identify potentially relevant studies, with keywords including “transcatheter mitral valve implantation,” “transcatheter mitral valve replacement,” “TMVI,” “TMVR,” “valve in valve,” “VIV,” “redo,” “mitral valve replacement,” and “SMVR,” until 15 September 2022.

### 2.2. Study selection and data extraction

The inclusion criteria were as follows: (I) failure of mitral valve bioprosthesis or mitral valvuloplasty ring; (II) available comparative information between TMVR (including valve-in-valve and valve-in-ring) and redo SMVR; (III) studies that reported the outcomes of the TMVR and redo SMVR groups. The exclusion criteria were as follows: (I) case reports, reviews, meta-analyses, animal studies; (II) duplicated publications; (III) conference abstracts without sufficient data.

Data were extracted by two investigators independently for the following variables: year of publication, study design, number of patients, patients’ sex, patients’ age, country, study period, in-hospital mortality, stroke, renal dysfunction, vascular complication, pacemaker implantation, exploration for bleeding, paravalvular leak, mean mitral valve gradient, 30-day mortality, and 1-year mortality. All discrepancies were resolved by seeking the opinion of a third reviewer or by consensus.

### 2.3. Risk-of-bias assessment

The Newcastle–Ottawa scale (NOS) was used to assess the risk of bias by two investigators independently. NOS was used to assess retrospective cohort studies. All disagreements between the two investigators were resolved by negotiated settlement. The results are shown in Table 1.

**TABLE 1:** Characteristics of the studies included in the meta-analysis.

References	Country	Patients (*n*)		Female (*n*)		Age (years)		Study period	Quality score	Design
		TMVR	Redo SMVR	TMVR	Redo SMVR	TMVR	Redo SMVR			
Zahid et al. (11)	USA	791	841	438	455	75 (M)	73 (M)	2015–2019	8	Retrospective cohort
Szlapka et al. (12)	Germany	79	79	47	46	74.7	72.2	2014–2019	8	Retrospective cohort
Simard et al. (26)	USA	86	129	54	81	74.9	64.5	2014–2020	7	Retrospective cohort
Gill et al. (13)	USA	310	310	160	190	73.0	72.0	2016–2018	8	Retrospective cohort
Liu et al. (27)	China	25	54	11	42	75 (M)	67.5 (M)	2013–2021	7	Retrospective cohort
Zubarevich et al. (28)	Germany	41	33	19	22	73.6	63.7	2012–2020	7	Retrospective cohort
Simonetto et al. (29)	Italy	49	29	30	13	77.6	67.7	2012–2019	7	Retrospective cohort
Kamioka et al. (30)	USA	62	59	38	36	74.9	63.7	2007–2017	7	Retrospective cohort
Murzi et al. (7)	Italy	21	40	13	23	77.0	67.0	2005–2015	7	Retrospective cohort

TMVR, transcatheter mitral valve replacement; Redo SMVR, redo surgical mitral valve replacement; M, median.

### 2.4. Statistical analysis

RevMan 5.4 (Cochrane; Oxford, UK) was used for statistical analysis. We used *I*^2^ to assess the heterogeneity of the included studies as follows: 25–49%, low heterogeneity; 50–74%, moderate heterogeneity; ≥ 75%, high heterogeneity. Random-effects models were used to assess summary estimates and 95% confidence intervals (CIs) for each outcome event. The odds ratios (OR) of all outcome events were meta-analyzed. If significant heterogeneity was found, sensitivity analyses were conducted, and *P* < 0.05 was considered statistically significant.

## 3. Results

### 3.1. Baseline characteristics

**FIGURE 1:**
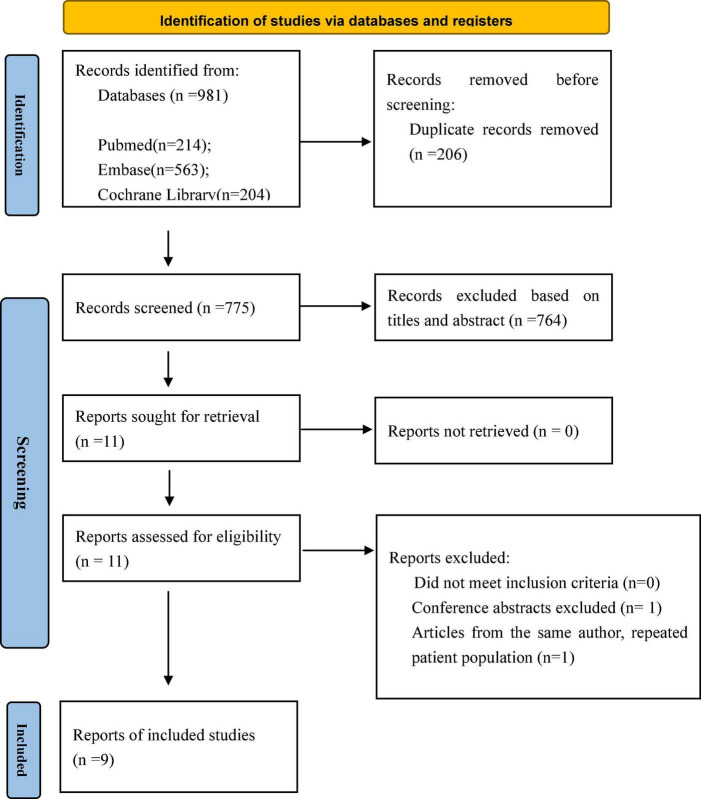
PRISMA flowchart showing the selection of studies for analysis.

A total of 981 potentially relevant publications were identified in the initial search. After removing the duplicates, 775 citations remained, and then, 764 publications were removed after screening the titles and abstracts. Next, 11 full-text articles were obtained and assessed in accordance with the predetermined inclusion and exclusion criteria. Ultimately, nine published articles were included in our meta-analysis. Figure 1 shows the flowchart of the study selection. The characteristics of the selected studies are listed in Table 1. The period of study was 2005–2021. All of the studies were retrospective cohort studies. Three studies used the propensity score matching method to reduce differences in baseline data (11–13). Patient characteristics are shown in Table 2. A total of 3,038 patients with mitral prosthesis failure undergoing mitral valve replacement were analyzed, including 1,464 patients with TMVR and 1,574 patients with redo SMVR.

**TABLE 2:** Patients characteristics.

References	NYHA III–IV, *n* (%)			STS score, %			TR ≥ moderate, *n* (%)			BMI (kg/m^2^)		
	TMVR	Redo SMVR	*P*	TMVR	Redo SMVR	*P*	TMVR	Redo SMVR	*P*	TMVR	Redo SMVR	*P*
Zahid et al. (11)	–	–		–	–	–	–	–		–	–	
Szlapka et al. (12)	3 (3.80)	4 (5.33)	–	15.7 (EuroSCORE II)	15.0 (EuroSCORE II)	0.533	32 (40.5)	29 (36.7)	0.174	–	–	
Simard et al. (26)	85 (98.8)	89 (69.0)	**0.0003**	–	–	–	41 (47.7)	78 (60.4)	0.07	27.1 ± 5.6	27.6 ± 6.5	0.56
Gill et al. (13)	–	–		–	–		–	–		–	–	
Liu et al. (27)	25 (100.0)	54 (100.0)	0.06	10.82 (7.72, 12)	3.36 (1.43, 5.23)	**< 0.01**	24 (96.0)	54 (10.0)	0.11	20.81 ± 2.39	22.95 ± 3.97	**< 0.05**
Zubarevich et al. (28)	41 (100.0)	24 (72.7)	–	11.9 ± 10.8	10.2 ± 14.3	**0.003**	25 (61.0)	16 (48.5)	0.35	26.4 ± 4.8	26.3 ± 4.3	0.59
Simonetto et al. (29)	42 (85.7)	16 (57.2)	–	8.7	3.6	–	15 (30.6)	4 (14.3)	–	24.4	24.2	–
Kamioka et al. (30)	–	–	–	12.7 ± 8.0	8.7 ± 10.1	**< 0.001**	39 (62.9)	32 (54.2)	0.33	–	–	
Murzi et al. (7)	18 (85.7)	29 (70.7)	0.25	39 ± 19 (EuroSCORE II)	23 ± 10 (EuroSCORE II)	**0.005**	2 (9.5)	4 (9.8)	0.653	–	–	

NYHA, New York Heart Association; STS, the Society of Thoracic Surgeons; EuroSCORE II, European System for Cardiac Operative Risk Evaluation II; TR, tricuspid regurgitation; BMI, body mass index. Bold values mean *P* < 0.05.

### 3.2. In-hospital mortality

**FIGURE 2:**
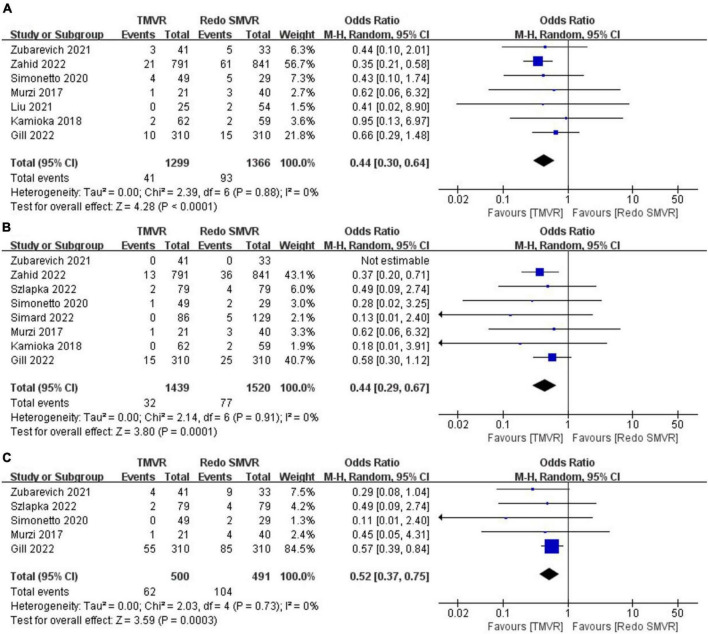
Forest plot comparing TMVR with redo SMVR for **(A)** in-hospital mortality, **(B)** stroke, and **(C)** renal dysfunction. CI, confidence interval; df, degrees of freedom; MH, Mantel–Haenszel.

Seven of the nine included studies reported in-hospital mortality. In total, 41 of 1,299 patients (3.2%) in the TMVR group died in hospital compared with 93 of 1,366 patients (6.8%) in the redo SMVR group. The OR for the comparison was 0.44 (95% CI: 0.30–0.64, *P* < 0.001; *I*^2^ = 0%, *P* = 0.88; Figure 2A), indicating that there was a statistically significant difference in in-hospital mortality between the two groups. Redo SMVR had higher in-hospital mortality than TMVR. *I*^2^ was 0%, which indicated low heterogeneity.

### 3.3. Stroke

Postoperative stroke was reported by eight of the nine articles. The merged outcome suggested that TMVR was associated with a lower stroke rate compared with redo SMVR (OR: 0.44; 95% CI: 0.29–0.67, *P* = 0.0001; *I*^2^ = 0%, *P* = 0.73; Figure 2B).

### 3.4. Renal dysfunction

Five studies reported the rate of renal dysfunction after the operation. When the random-effects model was used for the meta-analysis, we found that redo SMVR had a higher rate of renal dysfunction compared with TMVR. Moreover, there was a statistically significant difference (OR: 0.52; 95% CI: 0.37–0.75, *P* = 0.0003; *I*^2^ = 0%, *P* = 0.73; Figure 2C).

### 3.5. Vascular complication

**FIGURE 3:**
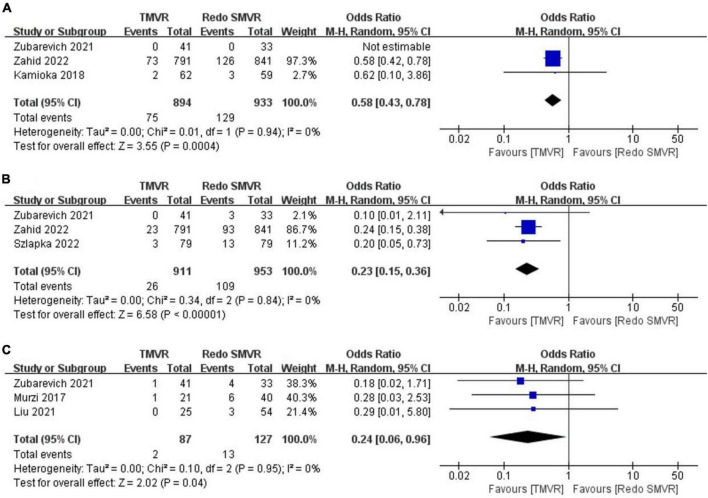
Forest plot comparing TMVR with redo SMVR for **(A)** vascular complication, **(B)** pacemaker implantation, and **(C)** exploration for bleeding. CI, confidence interval; df, degrees of freedom; MH, Mantel–Haenszel.

Data on vascular complications were available from three studies. After meta-analysis, TMVR was associated with a lower vascular complication rate than redo SMVR (OR: 0.58; 95% CI: 0.43–0.78, *P* = 0.004; *I*^2^ = 0%, *P* = 0.94; Figure 3A).

### 3.6. Pacemaker implantation

Pacemaker implantation rates were reported in three studies. Pooled analysis of outcome suggested that TMVR was associated with lower pacemaker implantation rates (OR: 0.23; 95% CI: 0.15–0.36, *P* < 0.00001; *I*^2^ = 0%, *P* = 0.84; Figure 3B).

### 3.7. Exploration for bleeding

In total, two out of 87 patients (2.3%) had an exploration for bleeding in the TMVR group compared with 13 of 127 patients (10.2%) in the redo SMVR group. TMVR was associated with a significant decrease in the risk of exploration for bleeding (OR: 0.24; 95% CI: 0.06–0.96, *P* = 0.04; *I*^2^ = 0%, *P* = 0.95; Figure 3C).

### 3.8. Paravalvular leak

**FIGURE 4:**
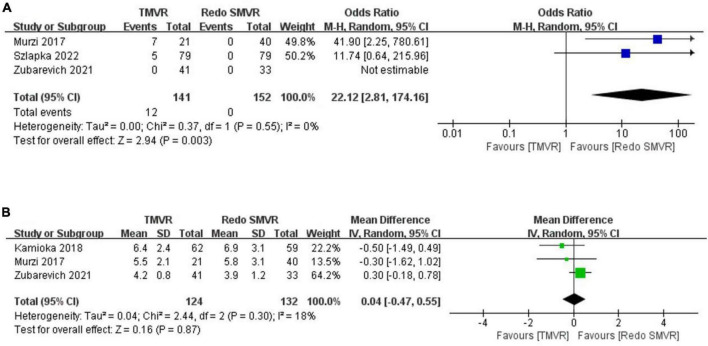
Forest plot comparing TMVR with redo SMVR for **(A)** paravalvular leak and **(B)** mean mitral valve gradient. CI, confidence interval; df, degrees of freedom; MH, Mantel–Haenszel; IV, inverse variance.

Postoperative paravalvular leak was reported in three studies. The rate of paravalvular leak was significantly greater in the TMVR group than in the redo SMVR group (OR: 22.12; 95% CI: 2.81–174.16, *P* = 0.003; *I*^2^ = 0%, *P* = 0.55; Figure 4A).

### 3.9. Mean mitral valve gradient

Three studies reported the postoperative mean mitral valve gradient. The pooled outcome suggested that there was no significant difference in the mitral valve gradient between the TMVR group and the redo SMVR group (MD: 0.04; 95% CI: −0.47 to 0.55, *P* = 0.87; *I*^2^ = 0%, *P* = 0.30; Figure 4B).

### 3.10. 30-Day mortality

**FIGURE 5:**
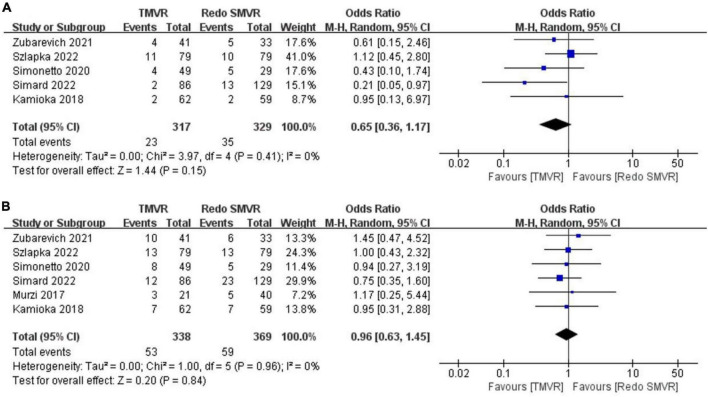
Forest plot comparing TMVR with redo SMVR for **(A)** 30-day mortality and **(B)** 1-year mortality. CI, confidence interval; df, degrees of freedom; MH, Mantel–Haenszel.

Data on 30-day mortality were available from five studies. There was no significant difference between TMVR and redo SMVR in 30-day mortality (OR: 0.65; 95% CI: 0.36–1.17, *P* = 0.15; *I*^2^ = 0%, *P* = 0.41; Figure 5A).

### 3.11. 1-Year mortality

Data on 1-year mortality were available from six studies. There was no significant difference between TMVR and redo SMVR in 1-year mortality (OR: 0.96; 95% CI: 0.63–1.45, *P* = 0.84; *I*^2^ = 0%, *P* = 0.96; Figure 5B).

## 4. Discussion

Mitral prosthesis failure represents a challenging therapeutic dilemma. The traditional and standard treatment is redo SMVR (14). However, redo SMVR is associated with an increased operative risk due to a number of factors, such as comorbidities and broad adhesions (14). For patients at a high surgical risk, TMVR is another viable treatment option (15). Following the development of transcatheter technologies in aortic valve replacement, transcatheter mitral valve-in-valve or valve-in-ring implantation has recently also been rapidly developing as an alternative to conventional surgical mitral valve redo procedures (16, 17). To date, the outcomes of both redo SMVR and TMVR therapy have been reported (5, 18). However, comparisons between redo SMVR and TMVR are limited. Therefore, we performed a meta-analysis to assess the outcomes of redo SMVR and TMVR for patients with mitral prosthesis failure.

In this meta-analysis of nine studies (3,038 patients), we found TMVR to be associated with lower rates of in-hospital mortality, stroke, renal dysfunction, vascular complication, pacemaker implantation, and exploration for bleeding, compared with redo SMVR. However, TMVR was associated with higher rates of paravalvular leak. There was no significant difference in postoperative mean mitral valve gradient, 30-day mortality, and 1-year mortality between the two groups.

In our study, in-hospital mortality was significantly higher in redo SMVR. Heterogeneity between studies was low (0%). All seven studies included in our pooled analysis showed a significantly increased in-hospital mortality rate in redo SMVR compared with TMVR. However, a previous meta-analysis that included only three articles (260 patients) showed no difference in in-hospital mortality between TMVR and redo SMVR (19). The reason for the discrepancy between the results of the two meta-analyses may be the small number of patients included in the previous meta-analysis and the lack of higher-quality studies. Although TMVR patients are older and have higher risk scores, in-hospital mortality is lower. This indicates that TMVR is safe and feasible to a certain extent. Gill et al. (13) reported that the only factor associated with higher mortality with TMVR was advanced kidney disease; in contrast, predictors of mortality unique to SMVR were age > 75 years, cirrhosis, sleep apnea, low body mass index, and obesity. Therefore, TMVR may also be a more suitable treatment for these patients.

Transcatheter mitral valve replacement was associated with a decreased incidence of vascular complications, despite having more vascular procedures. The physicians’ skillful puncture technique and the use of a vessel-closure device may be the reasons. In addition, TMVR was associated with lower rates of pacemaker implantation compared with redo-SMVR. The redo SMVR requires extensive debridement of the bioprostheses or reconstructive rings, whereas during TMVR, the failed bioprosthesis or ring may protect the conduction system from injury.

Our analysis suggested that patients with redo SMVR had a higher risk of stroke. Surgery performed under hypothermic ventricular fibrillation and retrograde perfusion through the femoral artery might be the factors associated with stroke after redo SMVR (7). Patients with redo SMVR had a higher risk of renal dysfunction. Patients with poor preoperative basic status combined with the influence of cardiopulmonary bypass are prone to renal dysfunction, and some patients need dialysis treatment. Of note, acute kidney injury is also considered a risk factor for death after surgery (20). In our report, SMVR was associated with a high risk of exploration for bleeding, which can be due to re-thoracotomy, large wound, long operation time, and difficulty in hemostasis. TMVR includes transapical and percutaneous approaches, both of which are less invasive than thoracotomy.

The incidence of perivalvular leakage was higher in patients with TMVR, which is consistent with findings reported in previous studies (12). Murzi et al. (7) reported the results of transapical TMVR versus redo SMVR; they showed that 28% of patients in the TMVR group had less than mild perivalvular leakage, compared with none of the patients in the SMVR group. These findings further suggest that for patients at an elevated risk of poor postoperative hemodynamics due to improper mitral valve position anatomy, redo SMVR may be the preferred intervention. Nevertheless, mild perivalvular leakage does not seem to have much of an adverse effect on the patients.

Redo surgery allows for the implantation of a bigger bioprosthetic valve. An elevated postoperative mean gradient can still be a limitation after a transcatheter valve-in-valve procedure, but transcatheter bioprosthetic valve fracture during TMVR offers a solution for patients with a small mitral bioprosthetic valve (21, 22). Interestingly, in our study, no significant differences were found in the mean mitral valve gradient between TMVR and redo SMVR. Hence, both procedures provide excellent and comparable hemodynamic results with low mitral valve gradients at follow-up. This indicates that TMVR does not affect the mitral valve gradient and does not cause hemodynamic abnormalities in patients. In this way, the long-term prognosis of patients can be guaranteed. Among the nine included studies, the implanted transcatheter valve included Sapien series and J-valve (Jiecheng Medical Technology, Suzhou, China), with predominance of Sapien. However, since the size of the largest prostheses on the market is only 29 mm, TMVR is not suitable for patients previously implanted with larger prostheses.

In our analysis, 30-day mortality and 1-year mortality were comparable between the two cohorts. Patients treated with TMVR can achieve comparable short-term outcomes to SMVR while reducing surgical trauma and the invasiveness of the procedure, especially in transseptal TMVR. Long-term follow-up results are needed to further confirm the effect of TMVR.

The current guidelines recommend concomitant tricuspid valve repair (TVR) in patients presenting with more than moderate tricuspid valve regurgitation (23). However, due to the lack of commercial transcatheter tricuspid products, concomitant TVR was performed only in the SMVR group. Although current guidelines recommend concomitant TVR, long-term outcomes of concomitant TVR in redo patients remain controversial (24, 25). A higher number of patients and longer-term follow-up are necessary to answer this question.

## 5. Study limitations

As the major limitation of this systematic review, all of the included studies were retrospective cohorts, which may reduce the value of this meta-analysis. In addition, this was a study-level meta-analysis; therefore, one relevant limitation is the lack of patient-level data. Furthermore, procedure bias or detection bias may have also influenced the outcomes of this meta-analysis. Thus, further studies, preferably in the form of randomized, large-scale, and strictly conducted trials, are needed to accurately evaluate TMVR in patients with mitral prosthesis failure.

## 6. Conclusion

Our results suggest that TMVR is effective at decreasing in-hospital mortality compared with redo SMVR in patients with mitral prosthesis failure. TMVR is also associated with lower rates of stroke, renal dysfunction, vascular complication, pacemaker implantation, and exploration for bleeding. Conversely, redo SMVR is associated with decreased paravalvular leak. There are no significant intergroup differences in postoperative mean mitral valve gradient, 30-day mortality, and 1-year mortality.

Transcatheter mitral valve replacement is a safe, feasible alternative to conventional redo surgery and may offer an effective and less invasive treatment for patients. Large randomized trials are necessary to elucidate the efficacy of TMVR as an alternative to redo SMVR for treating mitral prosthesis failure.

## Data availability statement

The original contributions presented in this study are included in the article/supplementary material, further inquiries can be directed to the corresponding author.

## Author contributions

JZ and YL conceived and designed the study. JZ and ZC collected and analyzed the data and wrote the manuscript. YL and HZ reviewed and edited the manuscript. All authors contributed to the article and approved the submitted version.
